# EXPLORING THE RELATIONSHIP BETWEEN INTERNET USE AND BELIEF IN CONSPIRACY THEORIES AMONG OLDER ADULTS IN JAPAN

**DOI:** 10.1093/geroni/igae098.2898

**Published:** 2024-12-31

**Authors:** Masumi Takeuchi, Ai Fukuzawa, Tetsuro Kobayashi

**Affiliations:** Tokyo Metropolitan Institute for Geriatrics and Gerontology, Tokyo, Japan; Musashino University, Nishitokyo, Tokyo, Japan; Waseda University, Tokyo, Japan

## Abstract

The use of the internet among older adults in Japan has become increasingly prevalent, leading to both positive and negative effects, such as the association between internet use and belief in conspiracy theories (Toriumi, 2023). In Japan, it has been observed that older adults are more susceptible to conspiracy theories when exposed to extreme content, such as online videos. However, there is a lack of empirical studies examining this phenomenon. This study aims to investigate the relationship between internet use and belief in conspiracy theories among older adults. An online survey was conducted in March 2024, involving 1,200 participants aged 20-79 residing in Japan. The survey assessed internet usage, belief in conspiracy theories, the UCLA Loneliness Scale, and basic demographic information. Participants were categorized into three age groups: young (20-39), middle-aged (40-59), and older adults (60-79). The results revealed no significant difference in belief in conspiracy theories among the age groups. However, multiple regression analysis indicated that among older adults, those who spent more time watching online videos were more likely to believe in conspiracy theories, particularly those who experienced higher levels of loneliness. No similar pattern was observed among the young and middle-aged participants. The results indicate that older adults, who have fewer social relationships due to retirement and other factors, may come to believe in conspiracy theories through viewing online videos. Further extensive research is required to establish protective measures that mitigate the potential risks associated with internet usage for the older population.

